# Cdk12 maintains the integrity of adult axons by suppressing actin remodeling

**DOI:** 10.1038/s41420-023-01642-4

**Published:** 2023-09-20

**Authors:** L. N. Townsend, H. Clarke, D. Maddison, K. M. Jones, L. Amadio, A. Jefferson, U. Chughtai, D. M. Bis, S. Züchner, N. D. Allen, W. Van der Goes van Naters, O. M. Peters, G. A. Smith

**Affiliations:** 1 School of Biosciences, Cardiff University, Cardiff, CF24 4HQ UK; 2 School of Medicine, Cardiff University, Cardiff, CF24 4HQ UK; 3 UK Dementia Research Institute, Cardiff University, Cardiff, CF24 4HQ UK; 4 John P. Hussman Institute for Human Genomics, University of Miami, Miami, FL USA; 5 Dr. John T. Macdonald Foundation Department of Human Genetics, University of Miami, Miami, FL USA

**Keywords:** Actin, Cellular neuroscience, Cell death in the nervous system, Cell death

## Abstract

The role of cyclin-dependent kinases (CDKs) that are ubiquitously expressed in the adult nervous system remains unclear. Cdk12 is enriched in terminally differentiated neurons where its conical role in the cell cycle progression is redundant. We find that in adult neurons Cdk12 acts a negative regulator of actin formation, mitochondrial dynamics and neuronal physiology. Cdk12 maintains the size of the axon at sites proximal to the cell body through the transcription of homeostatic enzymes in the 1-carbon by folate pathway which utilize the amino acid homocysteine. Loss of Cdk12 leads to elevated homocysteine and in turn leads to uncontrolled F-actin formation and axonal swelling. Actin remodeling further induces Drp1-dependent fission of mitochondria and the breakdown of axon-soma filtration barrier allowing soma restricted cargos to enter the axon. We demonstrate that Cdk12 is also an essential gene for long-term neuronal survival and loss of this gene causes age-dependent neurodegeneration. Hyperhomocysteinemia, actin changes, and mitochondrial fragmentation are associated with several neurodegenerative conditions such as Alzheimer’s disease and we provide a candidate molecular pathway to link together such pathological events.

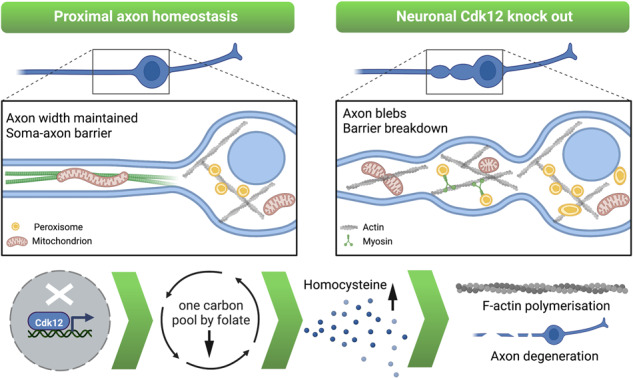

## Introduction

Cyclin-dependent kinases (CDKs) are regarded as key players in the development of the nervous system. Strick control of proliferation and differentiation of stem and progenitor cells comes from the complex orchestration of the CDKs, which ultimately define neuronal number and cell type. For example, genetic knockout either of cdk4 or cdk6 affects proliferation of basal progenitor cells [1] whereas inhibition of Cdk4 and CyclinD1 led to increased neurogenesis in the developing cortex [2]. Cdk12 is also required for the migration of late-arising cortical neurons by maintaining their genomic stability through expression of DNA damage response genes [3]. Cdk12, Cdk13, and Cdk5 have previously been found to contribute to axon elongation and patterning during development [4, 5] although the exact mechanism remains unclear. CDKs have therefore been strongly implicated in learning, memory, and synaptic plasticity [6, 7]. Mutations in these genes are linked several rare severe neurodevelopmental disorders in children who may present with lissencephaly, microcephaly, or developmental delay [8].

However outside of the developmental window the role that CDKs play remains uncertain, yet several remain highly expressed in terminally differentiated cells. More recent data suggest that they may a role beyond canonical cell cycle regulation to mediate specific pathways in adult neurons. CDK5 has been implicated as a conserved regulator of axon initial segment (AIS) size, acting locally to control microtubule organization and dynein-dependent trafficking through its activator p35 [9, 10]. The localization of CDKs in the nervous system may hold clues as to their function. CDK2 and CDK5 are localized to hippocampus neurons and sciatic nerves [11] where they function to control targeting of Kv1 channels to the axon. CDK14 is ubiquitously expressed and was found to play a role in axon regeneration after injury via Wnt signaling [12]. It has been hypothesized that in adult neurons nuclear resident CDKs may play a role in mediating neurodegeneration in response to DNA damage. In agreement with this, it was found that inhibition of CDK4 and CDK6 protect cortical neurons from the DNA damaging agent camptothecin [13]. The physiological functions of other CNS-enriched CDKs have yet to be found in the adult nervous system.

We identify a new function of Cdk12 in the maintenance of adult neurons in vivo. Cdk12 is a key upstream determinant of axon size in aged neurons, functioning to limit F-actin formation and dynamics in adult *Drosophila* axons. Ablation of this gene leads to an increase in F-actin formation, enlargement of axon in close proximity to the cell body and causes neurodegeneration during ageing. We find that Cdk12 normally functions to maintain the expression of genes associated with the catabolism of homocysteine (Hcy), which is likely the rate limiting step for F-actin dynamics. Cdk12 regulates actin patch formation in neurons, mitochondrial dynamics and neuronal polarity through the maintenance the axon-soma filtration barrier. We highlight the diverse roles of CDKs in adult neurons in vivo for neuronal compartmentalization, function, and survival.

## Results

### Cdk12 controls axonal size and neuronal physiology

We established a *Cdk12* mutant, first made by random mutagenesis in *Drosophila* using established methods [14–16]. The nature of this mutation was found to be deletion that resulted in a frameshift and premature stop codon. After several rounds of outcrossing and complementation studies the *Cdk12* mutation always mapped to lethality and can be classified as a null (Supplementary Fig. 1A). To circumvent lethality associated with the *Cdk12* mutation we investigated detailed age-dependent neuronal phenotypes using the mosaic analysis with a repressible cell marker (MARCM) clonal system, which utilizes genetically encoded fluorescent reporters to give single-cell resolution [17–19]. Homozygous clones are generated after mitosis bypassing early developmental effects linked to the cell cycle. Using this clonal system we were able to study the neuron-specific role of Cdk12 through its complete ablation in a small subset of neurons, while other neurons and cell types in the organism remained wild-type.

**Fig. 1:**
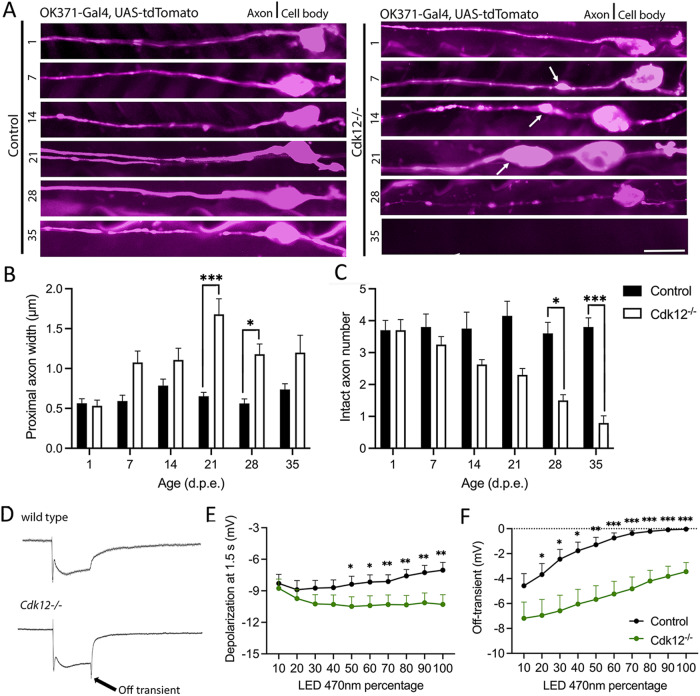
Cdk12 limits axon width specifically in the region closest to the cell body. **A** Mutations in the gene *Cdk12* caused an age dependent increase in proximal axon width and the formation of axonal swellings in wing sensory neurons. **B** *Cdk12*^−*/−*^ axon width was increased 2–3-fold at both 21 and dpe. **C** *Cdk12*^*−/−*^ axons began to degenerate at 28 days and a 75% loss of axons was recorded at 35 days p.e. **D** Example electroretinogram traces of wild type and *Cdk12*^*−/−*^ animals at 100% light intensity. **E** Loss of Cdk12 was associated with increased depolarization in response to 470 nm light. **F** Loss of Cdk12 caused a significantly greater off-transient response compared to control. Data was analyzed by two-way ANOVA and significant differences annotated as *p* < 0.05*, *p* < 0.01**, & *p* < 0.001*** between genotypes. White arrows indicate axonal swellings. Graphs are expressed as Mean ± SEM and *N* = ≥10 wings for each group. Scale bars = 10 µm.

Adult neuronal clones homozygous for the *Cdk12* mutation displayed a specific age-dependent swelling of the proximal axon region with the formation of large axonal blebs, and at later stages marked neurodegeneration was observed (Fig. 1A). At 21 days post eclosion (dpe), the primary axonal swelling in *Cdk12* mutated neurons was twice as wide as a wild-type axon (Fig. 1B) and by 35 dpe axons had degenerated at sites focal to the swellings in the proximal region (Fig. 1C). In contrast to axon phenotypes loss of Cdk12 did not cause morphological changes to the cell body (Supplementary Fig. 1B), but in 5% of cases caused the development of an additional dendrite (Supplementary Fig. 1C), suggesting that Cdk12 can modify the neuronal architecture in the somatodendrtic region. Importantly, re-expressing Cdk12 in neurons restored proximal axon width and rescued neurodegeneration (Supplementary Fig. 1D, E), confirming that Cdk12 can be considered a novel regulator of proximal axon size and maintenance.

The size of the proximal axon can impact on neuronal firing properties [20, 21]. In particular, this region is rich in ion channels required for computation and initiation of axon potentials and their modulation. We postulated that changes in proximal axon size might alter the properties of the AIS. Using the same EMS generated mutant and MARCM machinery, we therefore determined whether loss of Cdk12 at 8–14 dpe (prior to degeneration) would impact electrophysiology, through the use of electroretinograms (ERGs), where photoreceptor signaling in response to light is recorded in laminar neurons. Clonal loss of Cdk12 caused a significantly greater depolarization and off-transient response (Fig. 1D–F), while the on-transient and proportion of animals that returned to baseline following light stimulation was not significantly different (Supplementary Fig. 1F, G). This suggests that although neurons are effectively hyperpolarized in *Cdk12* mutant animals, photoreceptor depolarization is enhanced and consequently a greater repolarization event is required to return the neuron to a resting state. This ERG response is in contrast to the Trp channel mutant, which shows a greatly diminished depolarization and off-transient response, corresponding to lower calcium (Ca^2+^) levels [22, 23]. We therefore investigated whether in reverse *Cdk12*^*−/−*^ waveforms are associated with enhanced Ca^2+^. Using the cytosolic GCaMP6f indicator we found that loss of Cdk12 in neurons causes an increase in baseline Ca^2+^ (Supplementary Fig. 1H, I), which may explain evoked electrophysiological responses. A regional specificity to Ca^2+^ increases was observed, with strong GCaMP signal in the bleb region. Together these data indicate that Cdk12 controls axonal size and electrophysiology responses at early stages and prolonged loss of this gene results in neurodegeneration.

It has been shown that Cdk5 is localized to the AIS and is a key regulator of its length in vitro and in vivo [9, 10]. In contrast to Cdk12 depletion, proximal axon length is increased with Cdk5 overexpression [10]. We find that the overexpression of Cdk5 was not however associated with bleb formation or axon width changes in wild-type neurons and there was also no epistatic relationship of overexpressing or knocking down Cdk5 in *Cdk12*^*−/−*^ neuronal clones (Supplementary Fig. 2A). We also find in contrast to Cdk5, Cdk12 was not localized to the cytoplasm and was found exclusively in the nucleus (Supplementary Fig. 2B). This suggests that the roles of Cdks in proximal axon maintenance are diverse and their combined effect is to regulate width and length of this specialized sub-compartment through different intracellular mechanisms.

### Cdk12 regulates actin patches and dynamics in the proximal axon

**Fig. 2:**
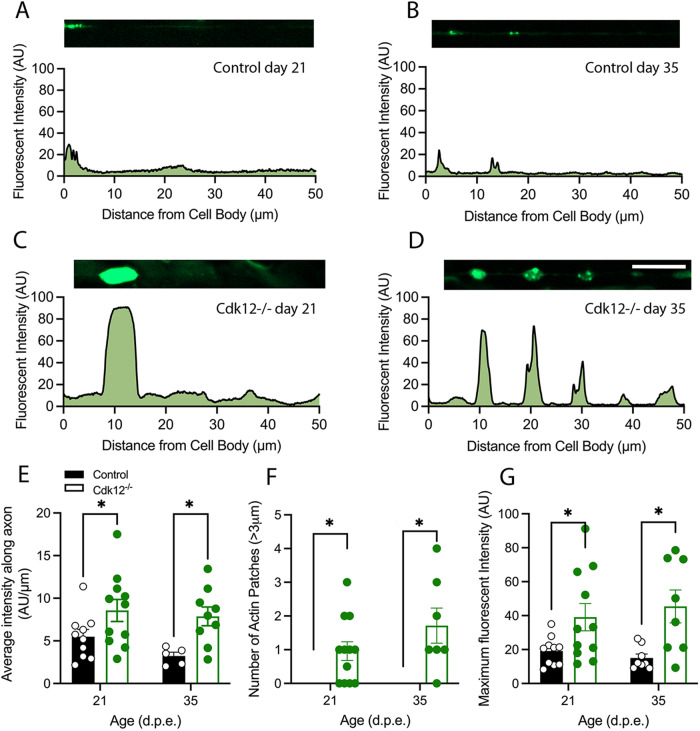
Cdk12 limits axonal b-actin patches. **A** A representative b-actin fluorescence intensity plot of small focal b-actin patches in wild type in wing sensory neurons at 21 days. **B** Aged wild-type axons at 35 days also display small patches of actin. **C** *Cdk12*^*−/−*^ axons display large axon patches that are intensely bright at 21 days. **D** By 35 days *Cdk12*^*−/−*^ axons the b-actin fluorescence intensity plot displays multiple bright axon b-actin patches up to 50 μm away from cell body. **E** *Cdk12*^*−/−*^ axons displayed a significantly greater area under the curve of fluorescent b-actin intensity plots. **F** Quantification shows that loss of Cdk12 is associated with increased b-actin patch formation. **G** The total fluorescence intensity of *Cdk12*^*−/−*^ axons was greater than control at both 21 days and 35 days. Fluorescence was normalized to td-Tomato expressed in the same cell to label membranes. Data was analyzed by two-way ANOVA and significant differences annotated as *p* < 0.05* & *p* < 0.01** between genotypes. Graphs are expressed as Mean ± SEM and *N* = ≥ 8 wings for each group. Scale bar = 10 µm.

Actin is enriched in the proximal axon and somato-dendritic regions to aid with ion channel localization, myosin myosin-dependent trafficking and is the key component of vesicle/ organelle barrier formation [24]. By expressing a genetically encoded marker for β-actin in *Drosophila* neurons we showed that actin is usually present in small distinct patches in the axon proximal to the cell body region and its size and location changes little as neurons age (Fig. 2A, B). Cdk12 ablation causes a significant enlargement of β-actin patches at young ages (Fig. 2C) and an increase in their frequency at later stages (Fig. 2D). β-actin patches were always closely associated with axonal blebs. Their increased frequency with age also co-insides with the onset of neurodegeneration. *Cdk12*^*−/−*^ neurons have significantly more actin across the length of the proximal axon (Fig. 2E), develop a greater number of patches (Fig. 2F), and have more β-actin overall (Fig. 2G). This suggests that age-dependent actin patch formation in *Cdk12*^*−/−*^ axons precedes proximal axon bleb development indicating an overall enlargement of the actin-rich somato-dendritic region.

**Fig. 3:**
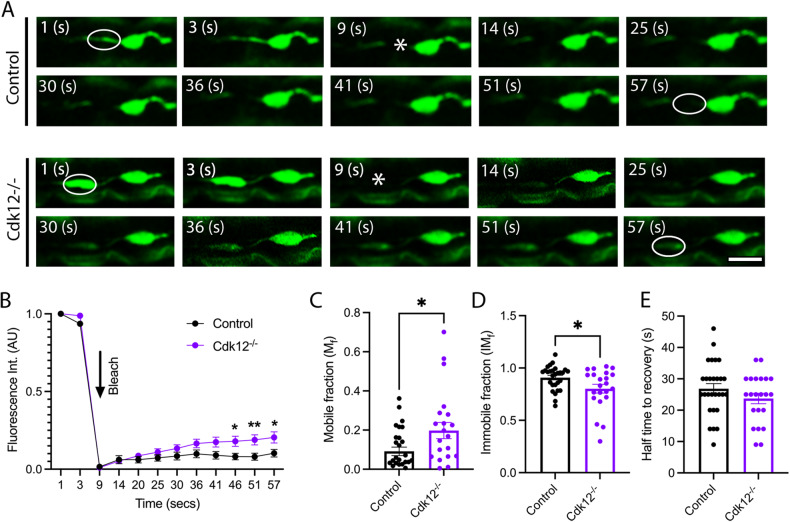
Cdk12 controls b-actin motility in the proximal axon. **A** FRAP experiments at 21 days in wing sensory neurons show that b-actin fluorescence at the region of the axon proximal to the cell body shows minimal recovery at 57 s post bleach, whereas recovery was observed in *Cdk12*^*−/−*^ axons. **B** maximum fluorescence recovery and plateau was seen at 46 s in all axons and a greater recovery was observed in *Cdk12*^*−/−*^ axons. **C** *Cdk12*^*−/−*^ axons display larger fraction of mobile b-actin compared to control. **D** *Cdk12*^*−/−*^ axons have less immobile b-actin compared to control. **E** No difference in the time to recovery was observed between genotypes. Data was analyzed by two-way ANOVA or T-test and significant differences annotated as *p* < 0.05* & *p* < 0.01** between genotypes. The region of interest for FRAP experiments are indicated by the white circle. Graphs are expressed as Mean ± SEM and *N* = ≥ 20 wings for each group. Scale bar = 5 µm.

β-actin is highly dynamic in neurons and undergoes coordinated assembly, disassembly, and treadmilling. A highly dynamic fast actin pool can be detected in neurons with a time constant less than a minute, while other species take 17 min or more to recover [25]. We next asked whether Cdk12 affects fast β-actin dynamics. Fluorescence recovery after photobleaching (FRAP) experiments further showed that fast pools of β-actin molecules are more mobile in *Cdk12*^*−/−*^ neuronal clones than in wild-type (Fig. 3A). By 57 s post bleaching β-actin recovery is minimal in control neurons; however recovery is observed to a significantly higher level when Cdk12 is ablated (Fig. 3A). Loss of Cdk12 causes a significantly greater recovery of β-actin from 46 s to plateau at 1 min (Fig. 3B). *Cdk12*^*−/−*^ neurons thus have an increased fraction of mobile β-actin and a decreased immobile fraction (Fig. 3C, D), however the half time to recovery is not affected (Fig. 3E) suggesting that β-actin mobility rate is not changed. This suggests that enhanced mobility in *Cdk12*^*−/−*^ neurons is due to an overall greater number of diffusible species available to fill the bleached area.

### Cdk12 enhances actin filament formation

**Fig. 4:**
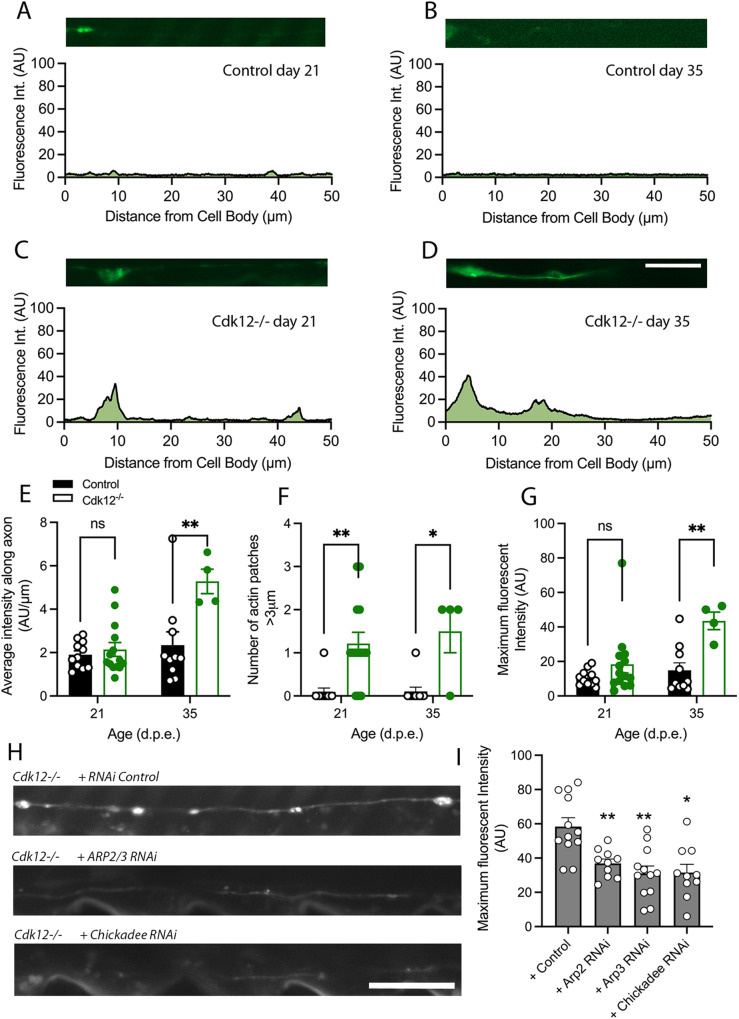
Cdk12 limits F-actin formation in axons. **A** A representative F-actin fluorescence intensity plot of F-actin localization in wild type in wing sensory neurons at 21 days using genetically encoded LifeAct. F-actin is highly expressed in small patches closer to the cell body. **B** Aged wild-type axons at 35 days also display F-actin in proximal axon regions. **C** *Cdk12*^*−/−*^ axons display large an increase in F-actin intensity at 21 days compared to age-matched controls. **D** By 35 days remaining *Cdk12*^*−/−*^ axons display an F-actin fluorescence intensity plot that shows multiple bright axon F-actin patches up to 30 μm away from cell body. **E** *Cdk12*^*−/−*^ axons displayed a significantly greater area under the curve of fluorescent F-actin intensity across 50 μm of axon. **F** Quantification shows that loss of Cdk12 is associated with increased F-actin patch formation. **G** The total fluorescence intensity of *Cdk12*^*−/−*^ axons was significantly greater than control at 35 days. Fluorescence was normalized to td-Tomato expressed in the same cell to label membranes. **H** Chickadee and the Arp2/3 complex were required for F-actin formation **I** RNAis targeted against actin-binding proteins rescued the heightened actin phenotype. Data was analyzed by either one or two-way ANOVA and significant differences annotated as *p* < 0.05* & *p* < 0.01** between genotypes. Graphs are expressed as Mean ± SEM and *N* = ≥ 8 per group. Scale bars = 10 µm.

Since FRAP experiments indicated that loss of Cdk12 may cause a more rapid processing of G-actin into F-actin, we next investigated the abundance and localization of specific F-actin species in the axon. Previous work using platinum replica electron microscopy has revealed that the proximal axon normally contains an enrichment of short and stable F-actin and longer dynamic F-actin species likely needed for diffusion barrier formation between the axon and the cell body [26]. In *Drosophila* neurons the expression of genetically encoded Lifeact shows that F-actin is also enriched in the axon-soma boundary (Fig. 4A, B). Loss of Cdk12 leads to an increase in F-actin formation, which is enhanced by age (Fig. 4C, D). *Cdk12*^*−/−*^ neuronal clones have a greater coverage of F-actin in the proximal axon (Fig. 4E), an increased number of F-actin foci (Fig. 4F) and a greater overall fluorescence intensity (Fig. 4G). Thus in physiological conditions Cdk12 is not only necessary to limit the total b-actin pool in the axon proximal region of neurons but also attenuates F-actin formation.

Actin polymerization, depolymerization branching, and nucleation depend on the activity of several actin-binding partners [27–29] and we therefore investigated for their importance for axonal swelling formation in the Cdk12 depleted AIS. We found that RNAi-mediated knockdown of either Chickadee (Profilin), Arp2 or Arp3 resulted in a complete rescue of axonal swellings in *Cdk12*^*−/−*^ neuronal clones (Fig. 4H, I), demonstrating that regulated F-actin formation and branching is a key determinant in regulating axon size proximal to the soma.

### Cdk12 regulates axonal organelle dynamics and position

**Fig. 5:**
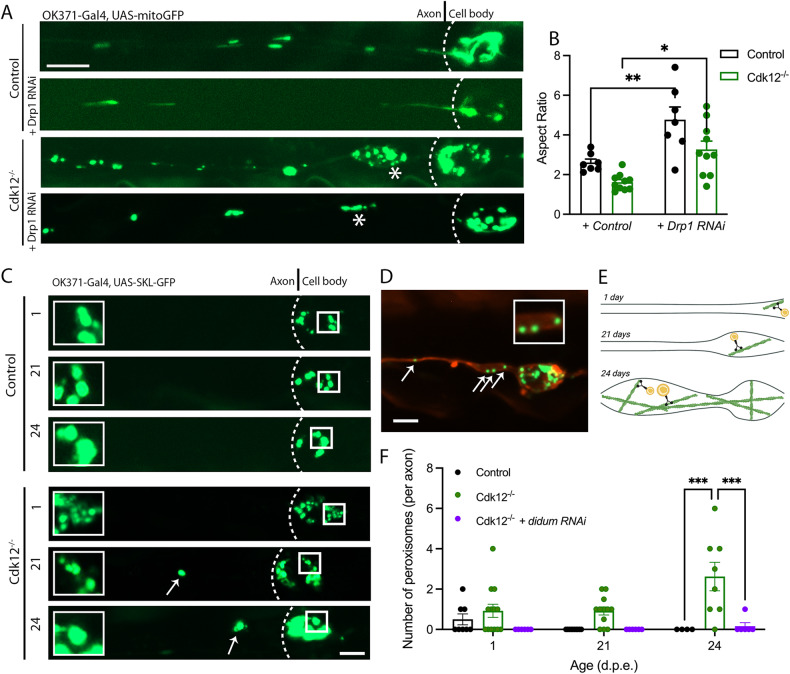
Cdk12 controls mitochondrial morphology and peroxisome position. **A** Mitochondrial morphology was observed at 21 days in wing sensory neuronal clones. *Cdk12*^*−/−*^ neurons and axonal swellings contain more spherical mitochondria compared to control, which was corrected with RNAi-mediated knockdown of Drp1 in axons. **B** Quantification shows that knockdown of the mitochondrial fission factor Drp1 caused an increase in mitochondrial aspect ratio in both wild-type and *Cdk12* ablated axons. **C** Peroxisomes are largely confined to somato-dendritic regions in wild-type neurons at 1, 21, and 24 days, yet knock out of *Cdk12* permitted age-dependent peroxisome entry into the proximal axon. **D** Peroxisomes in *Cdk12*^*−/−*^ axons were present in axonal swellings and non-swollen regions at 21 days. **E** Illustration to show that peroxisomes may be permitted to enter *Cdk12* ablated and aged axons on actin filaments via attachment to myosin motor proteins. **F** Quantification shows that at 24 days there are significantly more peroxisomes present in *Cdk12*^*−/−*^ axonal clones compared to control, which can be rescued via RNAi-mediated knockdown of didum (Myosin V). Data was analyzed by two-way ANOVA and significant differences annotated as *p* < 0.05*, *p* < 0.01**, & *p* < 0.001*** between genotypes. Dashed lines define the soma-axon boundary, * define axonal swelling regions and arrows highlight axon localized peroxisomes. Graphs are expressed as Mean ± SEM and *N* = ≥ 8 wings for each group. Scale bars = 5 µm.

The actin cytoskeleton plays an important role in mitochondrial network maintenance, including mitochondrial fission [30, 31], short range motility [32], and anchoring [33]. Therefore, the actin changes associated with loss of Cdk12 may ultimately impact on local mitochondrial dynamics. To test this, we used a genetically encoded fluorescent mitochondrial marker to investigate morphology in the proximal axon region of neuronal clones. Loss of Cdk12 caused the formation of round mitochondria at 21 days and their fragmentation was most abundant in axon swellings (Fig. 5A). Morphological changes in mitochondria manifested through a Drp1-mediated fission mechanism, since knockdown of this gene rescued the fragmented phenotype (Fig. 5A). Knocking down Drp1 using RNAi caused a significant increase in average mitochondrial aspect ratio in both WT and *Cdk12*^*−/−*^ clones, with a full rescue observed in the latter (Fig. 5B). The absence of Cdk12 in neurons also caused a decrease in the average area of individual mitochondria, a decrease in their Feret diameter and an overall increase in the number of mitochondria residing in the proximal axon stretch (Supplementary Fig. 3A–C). Drp1 was found to be the core determinant in mediating the mitochondrial changes observed (Supplementary Fig. 3A–C). In non-neuronal cells, Drp1-mediated fission was found to cause calcium overload in mitochondria and promote deficits in F-actin dynamics [34]. Interestingly, enhanced mitochondrial fission was not found to be the cause of axonal swellings nor neurodegeneration at latter stages (Supplementary Fig. 3D, E). We further investigated whether mitochondria were buffering enhanced axonal Ca^2+^ levels. Mitochondrial Ca^2+^ was not affected by increased cytosolic levels (Supplementary Fig. 3F, G), further suggesting that Ca^2+^ overload was not the cause of neurodegeneration. This demonstrates that Cdk12 normally functions to repress mitochondrial fission in neurons and that changes in actin dynamics likely occur upstream of Drp1 regulatory pathways. Neurodegeneration caused by ablation of Cdk12 is likely caused directly through heightened actin formation rather than aberrant mitochondrial dynamics.

Mitochondria are metabolically linked to peroxisomes which are responsible for the production of Acetyl-CoA esters through β-oxidation needed to fuel oxidative phosphorylation. Peroxisomes can associate with both microtubules and actin networks depending on cell type and animal species [35]. In adult *Drosophila* glutamatergic neurons we find peroxisomes are exclusively localized to the actin-rich somato-dendritic regions and this compartment is likely to be the primary point for β-oxidation to occur (Fig. 5C). This tight regulation of peroxisome position remains unchanged in the aged neuron. However, ablation of *Cdk12* caused peroxisomes to enter the proximal axon in an age-dependent manner (Fig. 5C), at time points correlating to the formation of axonal actin patches. Mislocalized peroxisomes were found predominantly within the proximal axonal swellings from 21 days (Fig. 5D). Peroxisomes present in *Cdk12*^*−/−*^ axons were also larger and more elongated in aged animals (Supplementary Fig. 4A–C). Interestingly, the modulation of peroxisome mass and shape by Cdk12 was not exclusive to axons and similar changes were observed in the actin-rich somato-dendritic region (Supplementary Fig. 4D–G). Peroxisome transport has been used to determine F-actin directionality in neurons where myosin-dependent movements correspond to actin patch length [36]. Given the role of Cdk12 to maintain actin in neurons, we next investigated whether peroxisomes were trafficked into the axon via actin-associated myosin motors. Knockdown of the myosin V ortholog didum caused a complete rescue of peroxisome mislocalization in *Cdk12*^*−/−*^ clones (Fig. 5E, F), indicating that peroxisomes are permitted into the axon via transport mechanisms on newly formed actin filaments. This demonstrates that Cdk12 functions as a regulator of the actin fence serving as an important vesicle/organelle barrier between the cell body and the axon. Excessive accumulation of F-actin in this region is likely orientated with plus (barbed) ends pointing toward the axon allowing for myosin-dependent transportation and barrier breakdown.

### Transcriptional regulation of genes in the one-carbon pool by folate pathway by Cdk12

**Fig. 6:**
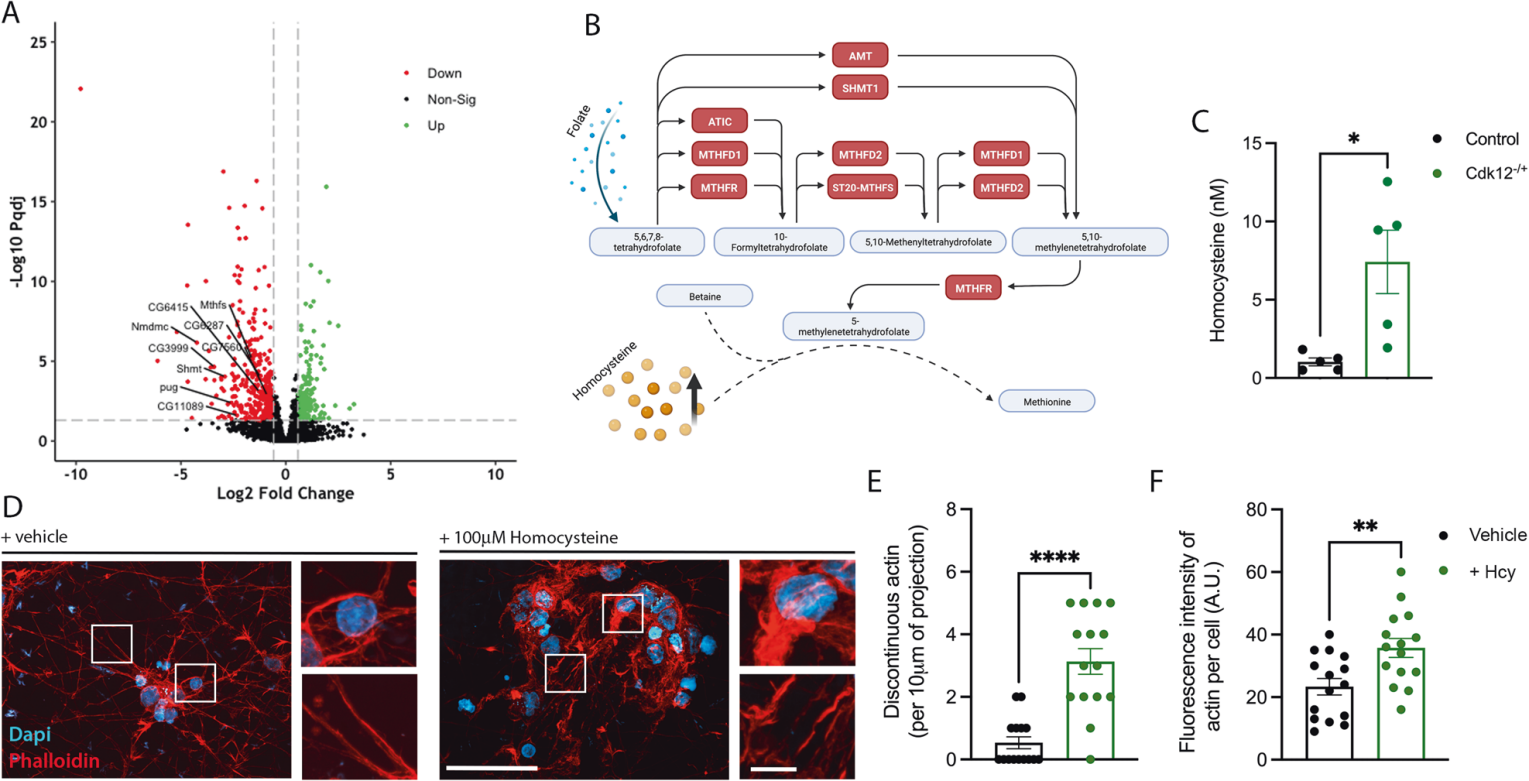
Cdk12 controls transcription of genes in the one-carbon by folate pathway, limiting homocysteine levels to allow for actin remodeling. **A** A volcano plot showing differentially expressed genes from head tissue of *Cdk12*^*-/+*^ animals compared to wild-type controls. Pathway analysis revealed an enrichment of genes in the one-carbon pool by folate pathway. **B** A schematic to illustrate that transcriptional downregulation of genes in the one-carbon pool by folate ultimately lead to elevated homocysteine levels. **C** Quantification shows that brains of *Cdk12*^*-/+*^ animals have elevated homocysteine (Hcy) levels at 21 days compared to age-matched wild-types. **D** Addition of Hcy to human iPSC-derived cortical neurons revealed significant changes in F-actin morphology. **E** Actin is more discontinuous in the projections with Hcy treatment. **F** Actin was enriched in the cell bodies with Hcy treatment. RNA sequencing data is expressed as log2 foldchange and significance found in a DESeq2 analysis with an FDR adjusted *P* threshold of 0.05, indicated by the dashed lines. T-tests were used to analyze levels of Hcy in *Drosophila* brains and actin changes associated with Hcy treatment. Graphs are annotated as *p* < 0.001***. Graphs are expressed as Mean ± SEM, *N* = 4 (*N* = 8 heads per replicate) for RNA sequencing experiments. 3 replicates were used for iPSC experiment, *N* = 15 images for quantification. Scale bars = 50 µm and inset = 5 µm.

Given the localization of Cdk12 to the nucleus we hypothesized that actin dynamics may be mediated by transcription mechanisms. Cdk12 is also required for the function of RNA polymerase for transcriptional elongation [37] and heterochromatin enrichment to influence gene expression [7]. We therefore performed RNA sequencing of *Drosophila* heads which revealed a number of differentially expressed genes—403 downregulated and 174 upregulated—in *Cdk12* heterozygous conditions (Fig. 6A). Data could be distinguished in 3 main principal components (PCs), with the first PC accounting for 41% of the data variation (Supplementary Fig. 5A). Genotype specific separation was observed in 2-dimensional analyses between PC1 and PC2 or PC3, but not between PC2 and PC3 (Supplementary Fig. 5B-D) and *Cdk12*^*+/-*^ and wild-type groups could be separated in space. g:Profiler pathway analysis of differentially expressed genes [38] revealed several dysregulated pathways including two prominent KEGG-associated metabolic pathways (Supplementary Fig. 5E). Surprisingly, we did not uncover any genes or pathways related to actin dynamics. Instead, we found several of the highly significant transcriptionally repressed genes associated with metabolism could be mapped to the one-carbon (1C) pool by folate pathway (Fig. 6A, B). The enzymes in this system are critical for the amino acid homeostasis of glycine, serine, and methionine, epigenetic maintenance and redox defense and can impact longevity in *C. elegans* [39]. Folate molecules function as carriers for 1C units which can be interconverted between different oxidation states. Specific conserved enzymes downregulated in our data CG7540 (MTHFR), CG6415 (AMT), pug (MTHFR1), Shmt (SHMT1) and Mthfs (ST20-MTHFS) show that all 1C species are likely to be reduced with the net effect being the accumulation of the homocysteine (Hcy) pool (Fig. 6B). Analysis of Hcy levels in adult fly brains revealed a significant increase of this amino acid in *Cdk12* heterozygous conditions (Fig. 6C). Hcy has found to be a regulator of actin dynamics in endothelial cells, astrocytes and cancer lines in vitro [40–42]. Our data suggest Hcy also regulates actin dynamics in adult neurons in vivo. To test this we applied Hcy to human iPSC-derived cortical neurons at a dose previously used in primary cells [41]. We find that Hcy caused F-actin disorganization in both projections and around the soma, including bleb and patch formation (Fig. 6D–F). These data suggest that Cdk12 normally functions to maintain enzymes in the 1C pool by folate pathway to prevent hyperhomocysteinemia in neurons. Excessive Hcy levels ultimately impact on F-actin dynamics in neurons to alter the localization and function of the axon-soma filtration barrier.

## Discussion

The canonical role of cyclin-dependent kinases (CDKs) is to regulate the cell cycle, however, they are also present in fully differentiated neurons that remain for an entire lifetime where their function is understudied. We place Cdk12 as a transcriptional repressor of homeostatic genes that control the balance of amino acid populations in neurons. Hcy levels are likely to control the amount of actin polymerization in the proximal axon. This work adds to the growing repertoire of CDKs to specifically regulate the size and function of neurons. It also demonstrates the importance of the actin cytoskeleton to prevent age-dependent neurodegeneration [43].

F-actin networks in the proximal axon are required to maintain the soma-axon filtration barrier to separate cargos and maintain polarity [44], where filaments are largely oriented with their plus ends facing toward the cell body [36]. We find that Cdk12-mediated enrichment of dynamic actin in the axon is organized, at least in part, since it allows for Myosin-V-mediated trafficking of cargos from the soma. This suggests that actin-binding partners may be involved in F-actin assembly. Large scale proximity assays are now helping to uncover new actin-binding partners located in the AIS such as Mical3 [45] and their function in barrier formation awaits investigation. More homeostatic upstream regulators that control this process, such as Cdk12, should also be considered. We also find that actin present at the beginning of the axon controls local mitochondrial dynamics, which may shed light on compartmentalized neuronal control of the network. Actin had been previously found to be localized to specific mitochondrial subpopulations [46], modulate mitochondrial dynamics [30, 47], trafficking, mitophagy [48], and metabolism [49] and control the formation of mitochondria-ER contact sites [50]. Our work further demonstrates that Drp1-mediated fission acts downstream of actin changes to alter mitochondrial morphology. However, this process may also underpin peroxisomal changes such as the increase in mass observed within the somato-dendritic region, warranting future investigation. It is therefore feasible that Cdk12 is an upstream determinant for energy capacity and organelle homeostasis in the axon.

Cdk12, Cdk13, and Cdk5 have previously been found to contribute to axon elongation and patterning during development [4, 5]. We suggest that this may in part be explained by the regulation of the actin cytoskeleton. Cdk5 was also found to be important for the maintenance of the organelle trafficking in the axon [9, 10]. However, our data shows that Cdk12 and Cdk5 are not epistatic in adult neurons, with each molecule having distinct roles for proximal axon homeostasis. Cdk12 is necessary to limit axon width in aged adult neurons in vivo, whereas Cdk5 modules AIS length, with both molecules impacting function. We find that at early adult stages loss of Cdk12 lead to electrophysiological dysfunction, which may underpin previous observations that Cdk12 is critically important for learning and memory [7]. Both heterochromatin remodeling on the X chromosome [7] and transcriptional changes across the whole genome likely play a role in neuronal maintenance. We uncovered a specific mechanism by which transcriptional repression of genes in the 1C by folate pathway cause a significant increase in the levels of amino acid Hcy, which contributes to actin dynamics in neurons where actin forms into disorganized patches [24]. Hcy has been shown to change actin dynamics in other cell types [40–42] although the functional consequence of this remains unclear. We now find that Cdk12 is a key regulator of actin dense regions of neurons in vivo that directly impact the function the vesicle/organelle barrier that separates the cell body from the axon. Mutations in actin or associated genes has been linked to several axonopathies including spinocerebellar ataxias, mental retardation, Baraitser–Winter syndrome 1, and juvenile-onset dystonia [51, 52]. Actin-related changes are also a common theme of several age-dependent neurodegenerative disorders [53]. Barrier breakdown may therefore also contribute to pathological state in these conditions.

Heightened levels of Hcy is well understood to promote excitotoxicity, DNA damage, Ca^2+^ overload, and apoptosis [54, 55]; we now include actin remodeling as an additional potentially catastrophic event in neurons. Under normal physiological conditions toxic Hcy species in the brain, produced by demethylation of methionine, are kept low through folate supplementation in the diet [56]. Neurons have an abundance of folate transporters [57] and its influx into cells can alter the G/F-actin ratio through cofilin dephosphorylation [58]. Interestingly, our results further show that loss of Cdk12 increases Ca^2+^ in the bleb region. This increase local increase Ca^2+^ itself may drive activation of actin-binding proteins such as profilin [59].

A multitude of epidemiological studies have found that folate deficiency during early pregnancy causes neural tube deficits during fetal development, due to decreased proliferation of both neuronal precursors and astrocytes [60]. The gene *MTHFR* (downregulated by Cdk12 ablation), was the first genetic risk factor to be identified for neural tube deficits [60], which indicates that high Hcy levels likely plays a key role in developmental disease prevalence. Evidence further supports elevated Hcy as a risk factor for age-dependent neurological conditions including Alzheimer’s disease (AD), where levels correlate with disease progression [61]. It is conceivable that Hcy may not just contribute to neurodegeneration through oxidative mechanisms, but also the hallmark symptoms that precede, such as cognitive impairment. In the AD patient brain cortical neurons that contain hyperphosphorylated tau showed changes in AIS length and position to more proximal regions within the axon [62], however, a more detailed investigation of actin in this region is much needed. AIS relevant changes in AD may in turn contribute to hyperexcitability and impaired hippocampal function. Cofilin-actin positive filaments are also seen in neurons close to amyloid beta (Aß) fibrils in vitro and in AD patient brains at post mortem [63]. We suggest that this may occur as a downstream event following heightened Hcy levels. Actin-dependent changes may also underpin the altered balance of mitochondrial fission and fusion found in AD [64, 65]. Interestingly Hcy itself can form aggregates that are present in close proximity to Aß plaques and could directly contribute to aggregation propensity [66]. Cdk12 may therefore be a viable therapeutic candidate that warrants investigation in AD model systems.

## Methods

### Drosophila stocks

*Drosophila* strains used to generate MARCM clones and for mutagenesis screening: *OK371-Gal4, 10xUAS-IVS-myr::tdTomato; FRT2A, FRT82B* crossed with *OK371-Gal4, 10xUAS-IVS-myr::tdTomato, asense-FLP2e; FRT2A, tub-Gal80* using methods and stocks previously described [14–17]. The following available *Drosophila* strains from Bloomington Drosophila Stock Center (BDSC) used for further genetic crosses and epistasis experiments: *ey-FLP* (37721), *5xUAS-Act5c::GFP* (8807), *5xUAS-LifeAct::GFP* (35544), *5xUAS-mito::GFP* (8442)*, 5xGFP-SKL* (28881)*, 5xUAS-Drp1*^*RNAi*^ (51483)*, 5xUAS-didum*^*RNAi*^ (55740), and *20xUAS-GCaMP6f* (42747) and *Df(3L)Exel9065* (7949). Further lines were obtained from Vienna Drosophila Resource Center (VDRC) to probe for genetic interactors with Cdk12: *5xUAS-Cdk5*^*RNAi*^ (35855)*, 5xUAS-twinstar*^*RNAi*^ (110599)*, 5xUAS-Arp2*^*RNAi*^ (101999 KK), *5xUAS-Arp3*^*RNAi*^ (53972) and *5xUAS-Chic*^*RNAi*^ (102759 KK)*. 5xUAS-Cdk5WT and 5xUAS-Cdk5KA were g*ifted by Dr Edward Giniger [5] and the *20xUAS-mito::GCaMP5* stock was generated previously [15]*. 5xUAS-Cdk12* and *5xUAS-Cdk12::GFP* plasmids were generated for rescue and labeling respectively experiments by standard sub-cloning procedures, using a *5xUAS, w*^*+*^ marker backbone and injected into embryos using the Phi31 integration service provided by BestGene Inc. 50% Males and 50% females used throughout and following genetic crosses and correct progeny collection were investigated at 1, 21, 24, or 35 days.

### Whole-genome sequencing

The underlying mutation in Cdk12 was discovered through the next-generation sequencing. Briefly, gDNA was extracted from ~200 heterozygous mutant adult flies and sequenced on the Illumina HiSeq2000 next-generation sequencing platform. Read alignment and genetic variant analysis was carried out at the Center for Genome Technology, University of Miami Miller School of Medicine.

### Visualization of wing neurons

*Drosophila* stocks were kept at 25 °C on standard cornmeal, molasses, and agar supplemented with dry yeast at 25 °C. The forward genetic screen was carried out according to [15]. Axonal MARCM clones were induced according to [17], so that neurons including any markers or reporters could be visualized in the L1 vein. Wings of anesthetized F1 flies used for screening and all quantitative imaging experiments (*N* = 5–20 per group), were cut close to the body using a dissection microscope (Zeiss), light source, and dissection scissors (EMS, VANNAS) and mounted on a microscope slide in Halocarbon Oil 27 (Sigma). A coverslip was placed on top and then used immediately for microscopy. Live cell imaging for quantification of cytosolic GCaMP and mito-GCaMP indicators was carried out according to, where the fly is restrained in a custom made chamber [67]. All microscopy experiments were carried out using a Zeiss Cell Observer spinning disk microscope using a 63x oil objective and Axiocam. Zen Blue software was used for both accusation and building time course and orthogonal projections. ImageJ (NIH) was used to measure the fluorescence intensity of actin using the ‘Integrated Density’ function. ImageJ was also used to analyze the morphology of peroxisomes and mitochondria in wing images using the line tool and ‘shape descriptors’ function, using the scale bar for pixel to length conversion. Mitochondria aspect ratio (AR) was quantified as the ratio between centerline length and average width, general area measured in μm^2^, and Feret’s diameter as the distance of two tangents to the contour of the particle in a defined orientation, useful for irregular shaped particles.

### FRAP

The Leica SP8 Confocal Microscope with Leica Application Suite (LAS)-X Core were used for FRAP experiments. Regions of interest (ROIs) were bleached using a 488 nm laser and 63x/1.4NA oil immersion objective for 5 s in actin-rich swellings at the distal tip of wings. A FRAP cycle of 1-min total duration was used; with 2 pre-bleached images taken every 1.295 s, 5 images during each bleach every 1.295 s 10 images taken post bleach every 5.295 s. Pre- and post-bleached images were imaged at the same laser intensity. Fluorescent intensity of ROIs throughout the time-lapse was measured in ImageJ. The immobile fraction was calculated by subtracting the post-bleached fluorescent intensity from the pre-bleached intensity (1 − *mobile fraction*). The mobile fraction was quantified as the remaining fraction.

### Electrophysiology

ERGs from live immobilized *Drosophila* (*N* = 10 females per genotype, 8–14 days old) were recorded as described previously [68] and [69], with Ag/AgCl wire electrodes (3″ × 10 mm) sheathed with glass micropipettes containing Beadle-Ephrussi ringer. An LED excitation light source (CoolLed - 470 nm) attenuated through a neutral density filter was used. LED output was regulated to provide different stimulus intensities in a single recording session. Signals were digitized at a 10 kHz rate. The baseline was monitored for 1 s before a 1 s pulse of light was introduced, the recording was then maintained for a further 3 s to assess baseline return. On-transient amplitude, depolarization at 1.5 s, off-transient amplitude and a binary metric regarding whether the baseline value was reached post-stimulus was extracted from each trace for further analysis using a custom MATLAB script.

### RNA sequencing

#### RNA extraction and sequencing

Whole *Drosophila* heads (*N* = 6–7 heads per replicate, 4 replicates per genotype) from 7-day-old female *Drosophila* were dissected for RNA isolation according to the RNAqueous-Micro kit (AM1931). Snap-frozen RNA samples were then sent to the Cardiff Genome Hub where they were subjected to quantity and quality control via Qubit 3.0 fluorometer and Agilent TapeStation. Library prep was performed using an Illumina TruSeq stranded mRNA kit, consisting of poly-A enrichment and TruSeq3 adapter ligation. Paired-end mRNA sequencing was then performed on an Illumina NextSeq500 at a depth of 6.5 million reads per sample with a fragment length of 76 base-pairs.

#### Pre-processing

Raw reads were assessed for quality using FastQC (0.11.8) and consequently poly-G tails and TruSeq3 adapters were removed using IlluminaClip (Trimmomatic). In addition, low-quality bases (phred < 3) at the start or end of a read, and bases with an average quality <15 within a sliding window of 4, in addition to reads shorter than 20 bases total, were removed using Trimmomatic. The *Drosophila* genome was constructed, and reads were mapped to their corresponding locations using STAR genomeGenerate and alignReads respectively. Finally, the number of successfully aligned reads belonging to individual genes were counted using featureCounts.

### Differential gene expression analysis

To enrich for protein coding transcripts, all non-coding RNAs, transposons, and unassigned features were removed from the raw counts using SARTools [70]. Subsequently, a DESeq dataset was compiled using DESeqDataSetFromMatrix (DESeq2) [71], with a main effect of genotype. Differential gene expression analysis by fitting a negative binomial distribution with parametric dispersion-mean relations and Wald significance testing with 5% FDR correction was then performed by DESeq2. Raw RNA sequencing data as well as feature counts for each sample are deposited on ArrayExpress (accession E-MTAB-12090).

To implement principal component analyses, variance stabilization transformations were performed on the DESeq dataset and distance matrices were calculated. Cluster dendrograms with corresponding heatmaps were computed using the pheatmap package and principal component analyses were performed using prcomp (R stats package). Volcano plots of log2 foldchange against adjusted *p*-values (padj) were plotted with a padj threshold of 0.05 and a log2 foldchange threshold of +/−0.58 (ggplot2, RStudio). All differentially expressed genes, independent of directional change, were inputted into g:Profiler for functional enrichment analysis to identify significantly associated KEGG pathways and gene ontology terms.

### Biochemical analysis of homocysteine

For homocysteine quantification 5 adult fly heads were dissected and pooled per group from required genotypes, snap-frozen on dry ice and stored at −80 °C. Fly heads were then homogenized, the supernatant extracted used immediately for a colorimetric human homocysteine ELISA Kit (Creative Diagnostics) according to the manufacturer’s instructions. At the final step, plates were incubated for 15 min at 37 °C. Standards and experimental samples were run in duplicate on a microplate reader (FLUOstar Omega) set to measure absorbance at 450 nM. Concentrations were calculated from the standard curve generated.

### Human iPSC-derived cortical neuronal culture

Kolf2 iPSCs were cultured in feeder-free conditions in mTesR medium on vitronectin-coated (1:100 in PBS, 0.5 µg/cm^2^) plates. Medium was changed daily (100%) and cells passaged using ReLeasR every 3–5 days. Cells were incubated in a humidified incubator at 37 °C and 5% CO_2_. iPSC colonies grown to ~60% confluency were treated with rock inhibitor (10 μM) for 1 h prior to single cell dissociation with Accutase (10 min). Cells were plated onto Matrigel-coated plates (1:100 in KO-DMEM) in mTesR medium plus Rock inhibitor (10 μM) and allowed to settle overnight. The next day cells were washed, three times with DPBS and medium changed to SLI medium which contains; Advanced DMEM/F-12 supplemented with 1% penicillin/streptomycin and Glutamax (ADF/PSG), and Neurobrew 21 (without Vitamin A), SB431542 (10 μM), LDN193189 (200 nM) and endo-IWR1 (1.5 μM). This is defined was day 0 (D0) and medium was changed daily until D8. At D8 cells were treated with Rock Inhibitor (10 μM) for 1 h and passaged using Accutase at a ratio of 1:3 onto fresh Matrigel-coated plates. Medium was changed to ADF/PSG supplemented with 2% NeuroBrew (without Vit A), which was replenished daily until neuronal progenitor cell (NPC) stage. At D16 NPCs were harvested by dissociation with accutase, counted frozen in 500 µl of Cryostor (CS10) at 6 × 10^6^ cells per vial.

For neuronal differentiation, NPCs were thawed and plated on Matrigel-coated T25 flasks in ADF/PSG medium supplemented with 2% NeuroBrew (without Vit A) and grown in a T25 for 4 days. Following this, FGF2 (20 ng/ml) was added to the medium to allow proliferation of NPCs for 4 days before plating for terminal differentiation [72]. Cells were plated onto nitric acid washed coverslips coated with PDL (100 μg/ml in 0.1 M Borate buffer) and Matrigel (1:50 in *ADF*). NPCs were dissociated using Accutase and plated at a density of 1 × 10^5^ cells/13-mm glass coverslips and cultured in SCM1 medium which contained; Advanced DMEM/F-12 supplemented with Neurobrew 21 (2%, with Vit A), PD0332991 (2 μM), DAPT (10 μM), Forskolin (10 μM), CHIR99021 (3 μM), GABA (300 μM), CaCl_2_ (adjusted to final medium concentration of 1.8 mM)), and Ascorbic acid (200 μM). Medium was replenished (50%) every other day for 7 days. Media was then changed to SCM2 which contained; Advanced DMEM/F-12 and Neurobasal-A (1:1) supplemented with Neurobrew 21 (2%, with Vit A), PD0332991 (2 μM), CHIR99021 (3 μM), CaCl_2_ (final [1.8 mM]), and Ascorbic acid (200 μM). Media was replenished (50%) every other day for 14 days.

Following terminal differentiation neurons were cultured for 24 h in base medium consisting of; Advanced DMEM/F-12 and Neurobasal-A (1:1) supplemented with Neurobrew 21 (2%, with Vit A), CaCl_2_ (final [1.8 mM]) and Ascorbic acid (200 μM). The next day DL-Homocysteine (100 μM) or PBS control (0.2%) was added for 24 h.

### Immunocytochemistry and phalloidin staining

IPSC-derived neurons were fixed with 4% paraformaldehyde (PFA) in PBS for 15 min at room temperature and washed 3 times with PBS. Cells were blocked for 1 h at room temperature with 3% normal goat serum and 3% bovine serum albumin followed by incubation at 4 °C overnight in primary antibodies (1:500 GFAP; Sigma G3893, β-III-Tubulin Sigma T8660). After 3 washes with PBS, cells were incubated at room temperature for 2 h on a rocker with secondary antibodies. All secondary antibodies conjugated to Alexa-594 were and diluted (1:400) in blocking buffer. Conjugated Phalloidin-568 probe was incubated in the dark at RT (5 μl/ml in PBS) for 20 min followed by 3 washes with PBS. Cells were counter-stained with Hoechst at 1:10,000 in PBS and mounted on microscope sides using immunomount.

### Statistical analysis

Statistical analysis unless otherwise stated was carried out using GraphPad Prism (9.0). Data was checked for normality using Kolmogorov–Smirnov tests. Subsequent tests and post hoc tests were chosen to determine significance at the alpha level of 0.05. Data was analyzed by, either, T-test, one-way ANOVA or two-way ANOVA that were 2-sided and significant differences annotated as *p* < 0.05*, *p* < 0.01**, *p* < 0.001***, & *p* < 0.001**** between genotypes and conditions. Graphs are expressed as Mean ± SEM. The number of samples used for each experiment can be found in corresponding figure legends.

### Supplementary information

Supplementary Figure legends

Supplementary Fig. 1

Supplementary Fig. 2

Supplementary Fig. 3

Supplementary Fig. 4

Supplementary Fig. 5

## Data Availability

RNA-sequencing data is freely accessible as specified in the Methods sections. The datasets supporting all other data in this article are also available from the corresponding author upon reasonable request.
