# Supplementary material for: Cdk12 maintains the integrity of adult axons by suppressing actin remodeling
Source: Cell Death Discov. 2023 Sep 20;9:348. doi: 10.1038/s41420-023-01642-4 (PMC10511712; doi:10.1038/s41420-023-01642-4)
Supplement: Supplementary file 1 — Supplementary Figure legends [file 41420_2023_1642_MOESM1_ESM.docx]

**Supplementary Figure 1. A null mutation in Cdk12 alters neuronal morphology, electrophysiology and baseline calcium levels.** A) The candidate gene mutation was discovered through whole genome sequencing and deficiency mapping. Loss of Cdk12 was found to be lethal. B) *Cdk12^-/-^* clonal neurons did not display any difference in cell body size at any age and were always similar to wild-type neurons. C) *Cdk12^-/-^* clonal neurons displayed a weakly penetrant phenotype of an additional dendrite. D) Axonal swellings present at 21 days in the *Cdk12^-/-^* group were rescued by UAS re-expression of the Cdk12 transgene. E) The neurodegenerative phenotype observed at 35 days in the *Cdk12^-/-^* group was rescued by UAS re-expression of the Cdk12 transgene. F) Electroretinogram recordings demonstrated on-transients were not significantly altered by loss of Cdk12 in the eye. G) The proportion of total recordings to return to baseline after light stimulation, defined as whether an individual trace was able to reach pre-stimulation baseline after the on transient within 5 s, were not significantly altered by loss of Cdk12. H) A cytosolic GCaMP indicator was expressed in neuronal clones and measured through live cell imaging to show that loss of Cdk12 caused increased calcium. I) Quantification shows that *Cdk12^-/-^* axons displayed a higher baseline calcium level compared to controls at 21 days. A 2-way ANOVA or t-test was used to analyze data and significant differences compared to control neurons annotated as p<0.05*. Arrows indicate dendrites. Graphs are expressed as Mean ± SEM, N=6-12 per group. Scale bars = 5µm.

**Supplementary Figure 2. Cdk12 does not genetically interact with Cdk5 and is localized to the nucleus.** A) Overexpression or RNAi-mediated knockdown of Cdk5 did not change axon size in wild-type neurons at 21 days and did not alter the axonal swellings associated with loss of Cdk12. B) Expression of a GFP tagged Cdk12 transgene revealed the Cdk12 is localized to the cell body and was not detected in axons. Data was analyzed by 2-way ANOVA and significant differences annotated as p<0.01** between genotypes. Graphs are expressed as Mean ± SEM and N= 6 wings for each group. Arrow indicates Cdk12 localization. Scale bar = 5µm.

**Supplementary Figure 3. Cdk12 controls mitochondrial morphology in axons by inhibiting fission.** A) Quantification shows that individual mitochondria were smaller in *Cdk12^-/-^* axons compared to wild-type axons at 21 days and size was rescued via RNAi-mediated knockdown of mitochondrial fission factor Drp1. B) Mitochondria were significantly less elongated in *Cdk12^-/-^* axons compared to control and was rescued with knock down of Drp1. C) Quantification shows that there were a greater number of mitochondria in *Cdk12^-/-^* axons compared to wild-type axons at 21 days and was rescued via RNAi-mediated knockdown of Drp1. D) Knockdown of Drp1 or didum did not rescue axonal swellings associated with Cdk12 ablation in axons at 21 days. E) Neurodegenerative phenotypes observed at 35 days were not rescued by knockdown of Drp1 or didum. F) Mitochondrial calcium was visualized using a mitochondrial targeted GCaMP in both *Cdk12^-/-^* and wild-type neurons. G) Quantification shows that fluorescent intensity of mitochondrial targeted GCaMP was not significantly different in *Cdk12^-/-^* axons. Data was analyzed by 2-way ANOVA or t-test and significant differences annotated as p<0.05* & p<0.0001**** between genotypes. Graphs are expressed as Mean ± SEM and N= 7-10 wings for each group.

**Supplementary Figure 4. Loss of Cdk12 alters peroxisome morphology in neurons.** A) The area of individual peroxisomes in *Cdk12^-/-^* axons is increased with age and quantification shows that peroxisomes were larger at 21 days compared to 1 day. B) Peroxisomes were more elongated in *Cdk12^-/-^* axons and had a larger Feret diameter at 21 days compared to 1 day. C) The aspect ratio of axonal residing peroxisomes in the *Cdk12^-/-^* group was not effected by age. D) The total area of peroxisomes in the cell body was significantly increased in the cell body of *Cdk12^-/-^* neurons compared to control. Quantification shows that at 21 days peroxisomal area was double that of controls. E) Peroxisomes in the cell body were more elongated and had a greater Feret diameter in *Cdk12^-/-^* neurons compared to control at 1 day and peroxisomes in wild-type axons became more elongated with age. F) The aspect ratio of peroxisome mass in the cell body did not differ with genotype or age. G) Schematic to illustrate the increase in peroxisomal mass and elongation in the cell bodies of *Cdk12^-/-^* neurons with age. Data was analyzed by either 1-way or 2-way ANOVA and significant differences annotated as p<0.05* & p<0.01** between groups. Graphs are expressed as Mean ± SEM and N= 5-12 wings for each group.

**Supplementary Figure 5. Differential gene expression associated with Cdk12 ablation highlights several dysregulated pathways.** A) A scree plot identifying that the differential gene expression variation of *Cdk12^-/+^* brains compared to controls can be explained by multiple principal components, with the first principal component (PC) accounting for 41% of the differences observed. B) A 2 dimensional analysis of PC1 against PC2 shows a clear separation of *Cdk12^-/+^* and control groups and can be used to distinguish genotypes. C) A 2 dimensional analysis of PC1 against PC3 shows a clear separation of *Cdk12^-/+^* and control groups. D) A matrix generated for profiles of PC2 against PC3 was not accounted for by genotype entirely. E) g:Profiler pathway analysis of up regulated and down regulated genes indicated a number of enriched pathways dysregulated in in heterozygous *Cdk12* knock out brains compared to controls. labels highlight significant metabolic pathways associated with genes residing in the one-carbon by folate pathway and linked pathways.
