# Supplementary figures and images for: Cdk12 maintains the integrity of adult axons by suppressing actin remodeling
Source: Cell Death Discov. 2023 Sep 20;9:348. doi: 10.1038/s41420-023-01642-4 (PMC10511712; doi:10.1038/s41420-023-01642-4)

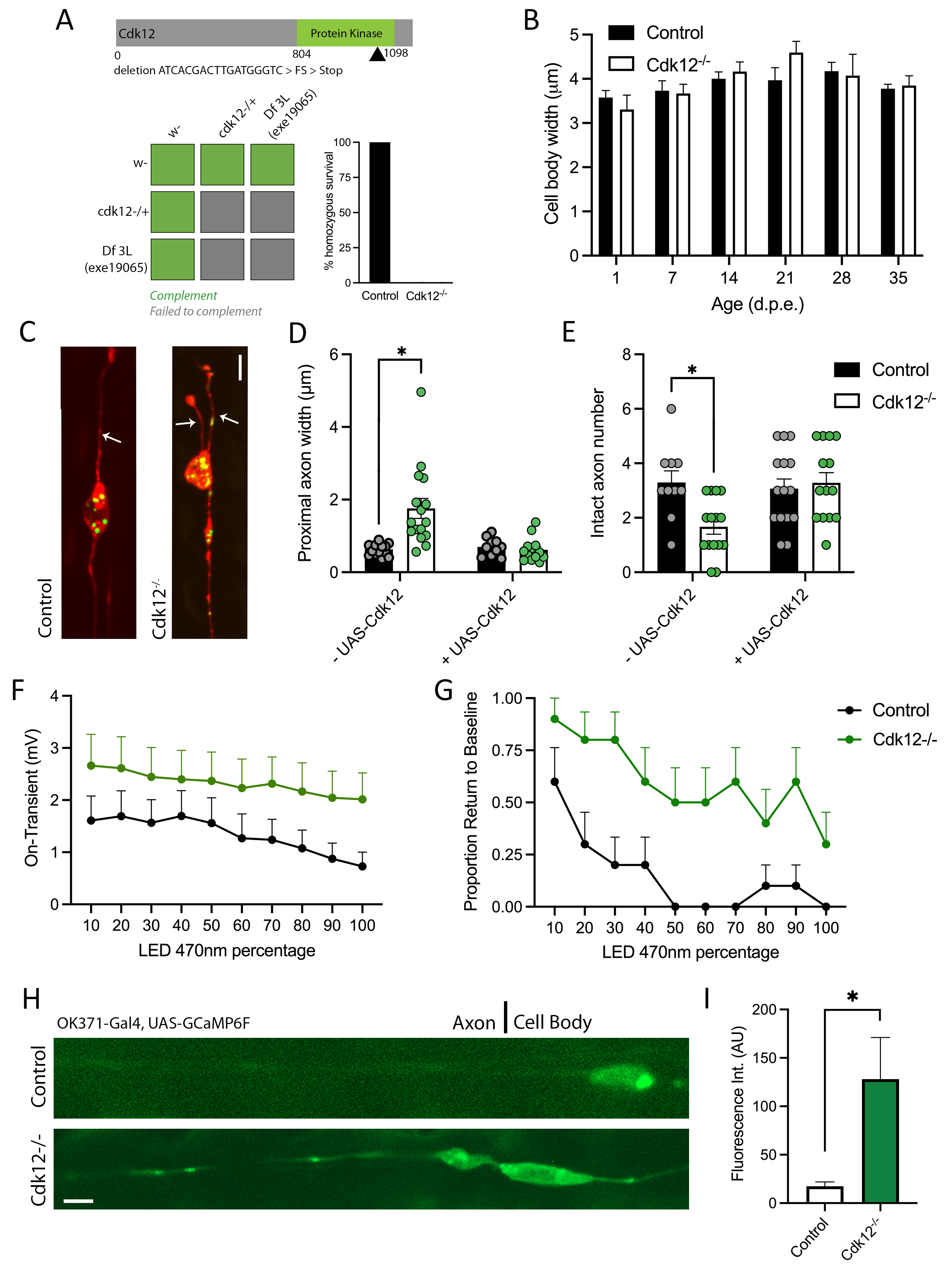

Supplement: Supplementary file 2 — Supplementary Fig. 1 [file 41420_2023_1642_MOESM2_ESM.tif]

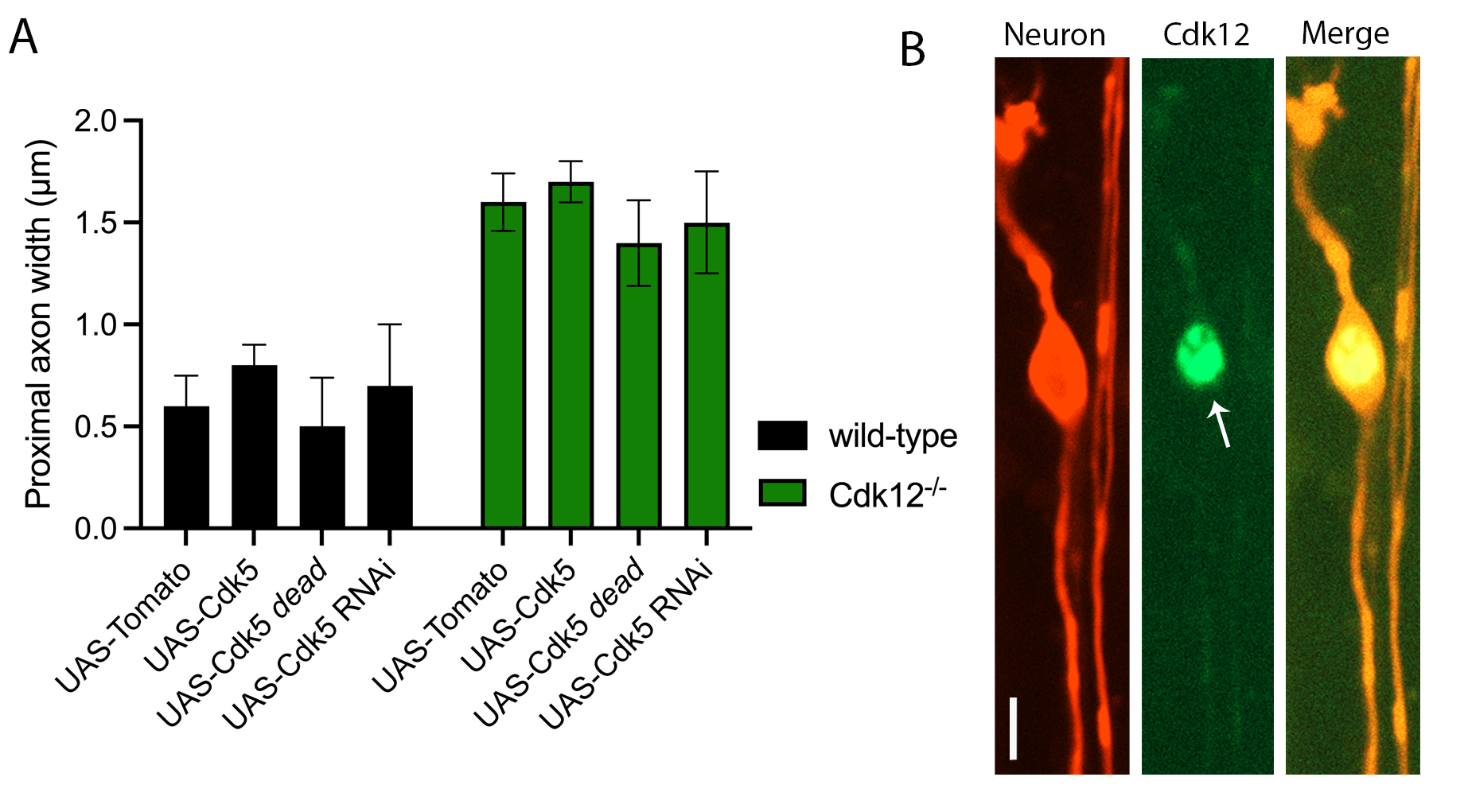

Supplement: Supplementary file 3 — Supplementary Fig. 2 [file 41420_2023_1642_MOESM3_ESM.tif]

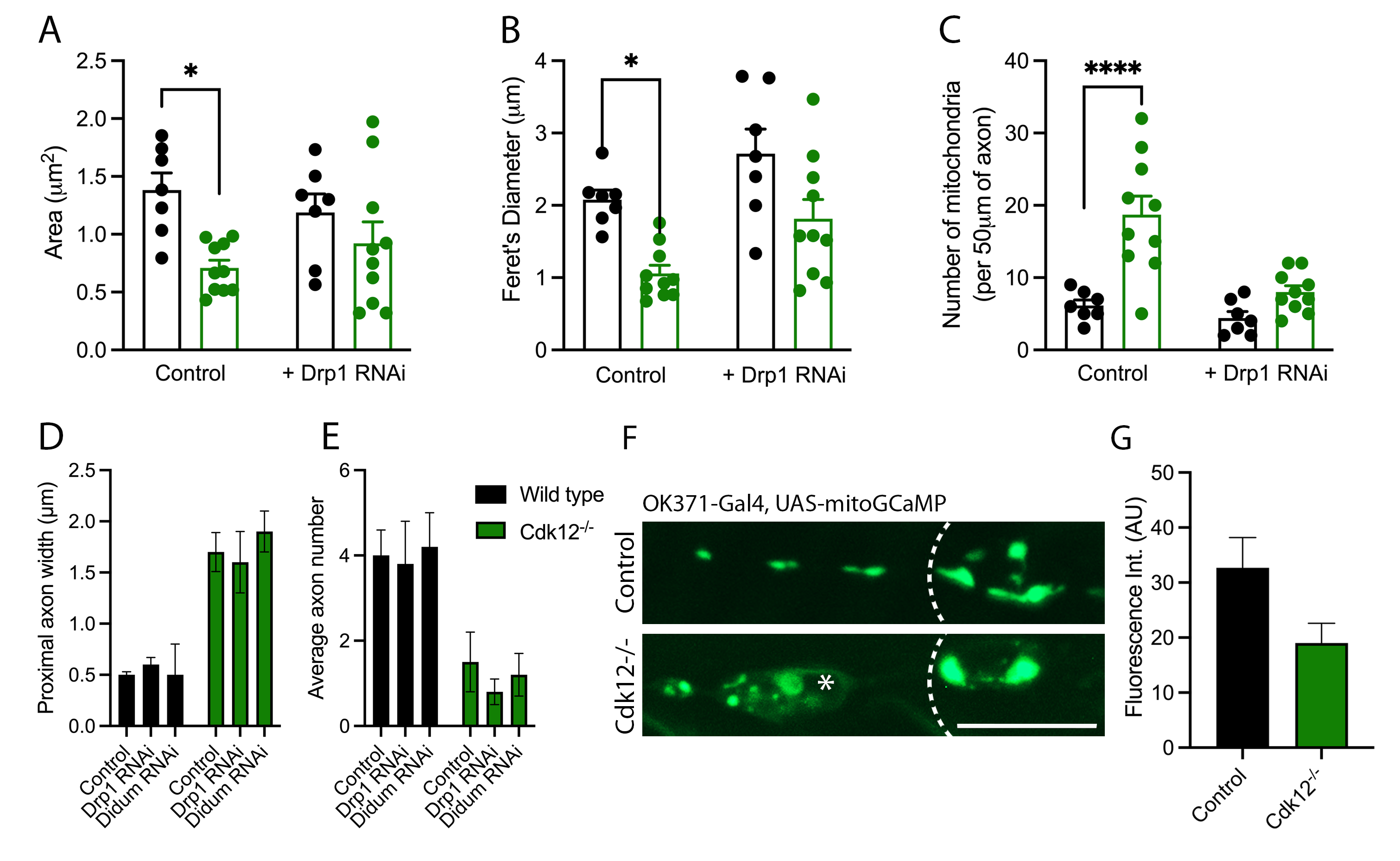

Supplement: Supplementary file 4 — Supplementary Fig. 3 [file 41420_2023_1642_MOESM4_ESM.tif]

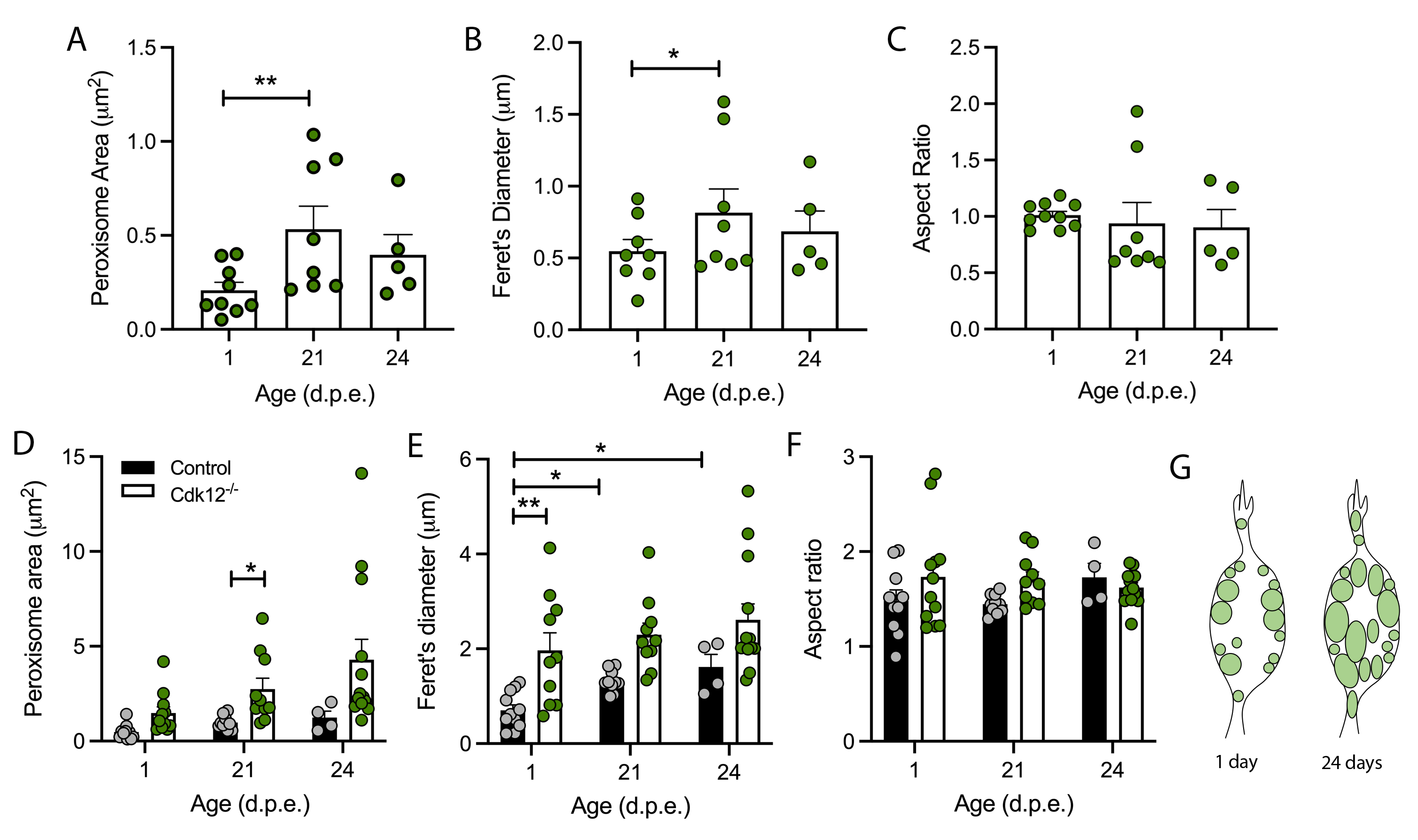

Supplement: Supplementary file 5 — Supplementary Fig. 4 [file 41420_2023_1642_MOESM5_ESM.tif]

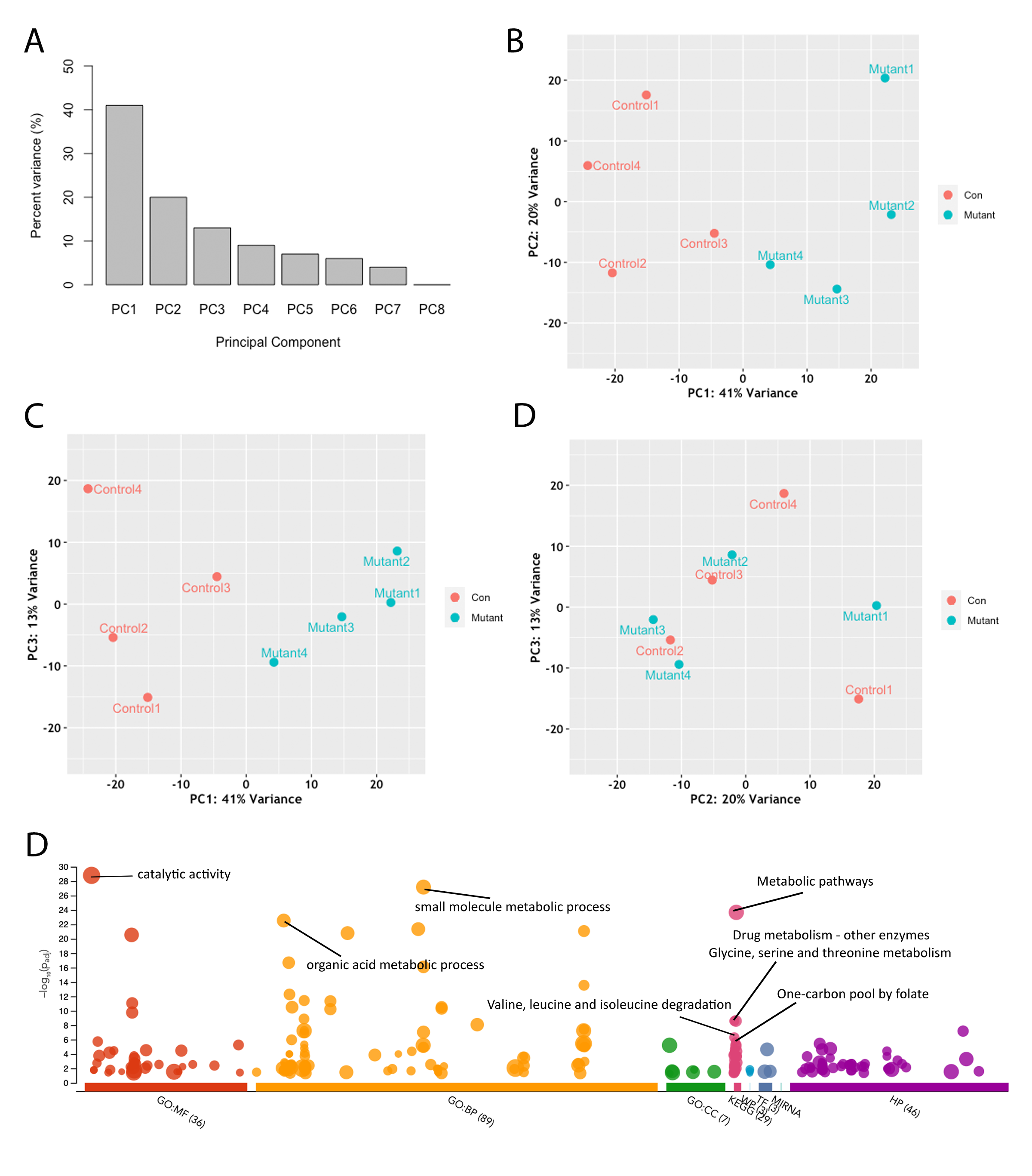

Supplement: Supplementary file 6 — Supplementary Fig. 5 [file 41420_2023_1642_MOESM6_ESM.tif]
